# LncRNA RWDD3 Facilitates Leydig Cell Steroidogenesis by Regulating the miR-1388-5p/NPY1R/cAMP Pathway in Yanshan Cashmere Goats

**DOI:** 10.3390/ani15131884

**Published:** 2025-06-26

**Authors:** Meijing Chen, Xuejiao Yin, Chunhui Duan, Yuchun Xie, Chenghao Ji, Yong Wang, Yueqin Liu, Yingjie Zhang

**Affiliations:** 1 College of Animal Science and Technology, Hebei Agricultural University, Baoding 071000, China; chenmeijing815@126.com (M.C.); duanchh211@126.com (C.D.); liuyueqin66@126.com (Y.L.); 2 College of Animal Science and Technology, Hebei Normal University of Science & Technology, Qinhuangdao 066000, China; bdyinxuejiao@foxmail.com (X.Y.); 15124796689@163.com (Y.X.); jichenghao1104@163.com (C.J.); 3 Hebei Key Laboratory of Specialty Animal Germplasm Resources Exploration and Innovation, Qinhuangdao 066000, China; 4 Inner Mongolia Academy of Agricultural & Animal Husbandry Sciences, Hohhot 010031, China; wangyongkeyan0322@126.com

**Keywords:** prolactin, ceRNA, testosterone synthesis, lncRNA, cashmere goat

## Abstract

Prolactin is a polypeptide hormone that plays a critical role in male reproduction. However, the underlying mechanisms of prolactin-regulated testosterone secretion and the roles of lncRNAs in this process remain unclear. We performed a comprehensive analysis of the testicular tissues of cashmere goats with different prolactin levels by means of RNA-sequencing. Then, we constructed a lncRNA–miRNA–mRNA interaction network by integrating previously submitted testicular mRNA sequencing data. We identified a novel lncRNA named lncRWDD3 and investigated its effects on testosterone synthesis in the Leydig cells of cashmere goat. The primary Leydig cells were used to explore the biological function of lncRWDD3/miR-1388-5p/NPY1R in vitro. This study found that 200 ng/mL of prolactin achieved the highest testosterone secretion in Leydig cells. LncRWDD3 or NPY1R overexpression promoted cAMP levels, testosterone secretion, and related gene expression, while lncRWDD3 or NPY1R interference had the opposite effect. It was found that lncRWDD3 acts on miR-1388-5p as a ceRNA, and neuropeptide Y receptor Y1 (NPY1R) was confirmed to be a target of chi-miR-1388-5p. Our research shows that prolactin regulates the testicular function of cashmere goats via the lncRNA–miRNA–mRNA ceRNA network, and lncRWDD3 acts as a ceRNA to activate NPY1R/cAMP signaling via the sponging of miR-1388-5p in order to govern testosterone synthesis in the Leydig cells of cashmere goats. Our results provide insights for future studies on the molecular mechanism of the prolactin regulation of testicular function in goats.

## Simple Summary

The underlying mechanisms of prolactin-regulated testosterone secretion and the roles of long non-coding RNAs (lncRNAs) in this process remain poorly understood. We performed a comprehensive analysis of the testicular tissues of cashmere goats with different prolactin levels via RNA sequencing and constructed an lncRNA–miRNA–mRNA interaction network. We identified a novel lncRNA named lncRWDD3 and investigated its effects on the testosterone secretion of goat Leydig cells. We found that 200 ng/mL of prolactin achieved the highest testosterone secretion and that lncRWDD3 acts on miR-1388-5p as a competing endogenous RNA (ceRNA). In addition, neuropeptide Y receptor Y1 (NPY1R) was proven to be a target of miR-1388-5p. Our study shows that prolactin can regulate testicular function through the ceRNA network and that the novel lncRWDD3 acts as a sponge for chi-miR-1388-5p to activate the NPY1R/cAMP pathway, thereby facilitating Leydig cell steroidogenesis.

## 1. Introduction

Prolactin (PRL) is primarily produced by lactotroph cells in the anterior pituitary gland [1]. Aside from its traditional role in lactation, prolactin is also a pleiotropic hormone that affects several physiological functions [2]. Accumulating evidence suggests that prolactin influences male reproduction. A study in rats revealed that high prolactin levels decrease the levels of follicle-stimulating hormone (FSH) and luteinizing hormone (LH) [3]. Clinical evidence indicates that hyperprolactinemia may lead to male infertility and reduced spermatogenesis [4]. Our previous study revealed that prolactin inhibition increases the serum concentrations of LH and testosterone and promotes the thickness of the spermatogenic epithelium and the diameter of seminiferous tubules [5]. However, the molecular mechanism underlying prolactin’s regulation of testicular function in male cashmere goats remains unclear.

LncRNAs are a class of recently discovered non-coding transcripts; they range in size from 200 bp to >100 kb and participate in various essential biological processes [6,7]. LncRNA has been discovered to play key roles in the regulation of testicular development and spermatogenesis in mammals [8,9]. A new hypothesis states that several lncRNAs could function as competing endogenous RNAs (ceRNAs) to protect mRNAs by acting as microRNA (miRNA) molecular sponges to specifically inhibit target mRNAs [10]. A previous study showed that lncRNA MIR22HG acts as a sponge for miR-125a-5p and prevents it from binding to NDRG2 3′UTR in Leydig cells, leading to NDRG2 transactivation, cell apoptosis aggravation, and testosterone synthesis inhibition [11]. In addition, another study showed that lncRNA LOC105611671 acts as a ceRNA by sponging oar-miR-26a to directly repress the expression of FGF9, which promotes testosterone biosynthesis [12]. The above evidence provides support for the study of the ceRNA regulatory network and its application to reproduction in male animals.

Although previous reports have preliminary shown that lncRNAs modulate the functions of Leydig cells, a comprehensive understanding of the concrete regulation mechanism and roles of lncRNAs is lacking, and it is unclear whether prolactin regulates testosterone synthesis in cashmere goat Leydig cells through a ceRNA regulatory mechanism. Therefore, in the present study, the influence of lncRNA regulation on testosterone synthesis was investigated in the Leydig cells of cashmere goats. We first constructed a ceRNA network (lncRNA–miRNA–mRNA network) that was connected to the prolactin regulation of testicular function. Then, we noticed that neuropeptide Y receptors (NPY1Rs), which belong to the superfamily of G-protein-coupled receptors (GPCRs), primarily activated or repressed the cAMP pathway, thereby participating in the testosterone synthesis regulated by lncRNA TCONS_00023611 (named lncRWDD3)/miR-1388-5p. Our results reveal that lncRWDD3 serves as a ceRNA that regulates testosterone synthesis in goat Leydig cells. This result reveals a new component of the physiological regulatory network of testosterone synthesis in testes and offers a vital experimental foundation for future studies on the reproductive function of male goats.

## 2. Materials and Methods

### 2.1. Animals and Experiments

Yanshan cashmere goats were used in this study. As described in our previous study [5], twenty healthy male goats (*Capra hircus*, Yanshan Cashmere goat breed, 10 months old, body weight = 22.98 ± 1.95 kg) were randomized into two groups: (1) a bromocriptine (BCR, prolactin inhibitor) treatment group (*n* = 10; 0.06 mg/kg BW) and (2) a control group (n = 10; equivalent water). The experiment lasted for 45 days. All of the goats were slaughtered on day 30 of the treatment. Portions of the testes were collected and stored.

### 2.2. RNA Sequencing (RNA-Seq) and Bioinformatics Analyses

Testis tissues from the two groups were used for RNA-seq. The total RNA was extracted using Trizol reagent. The RNA quantity and quality were detected, and the RNA integrity was further examined via agarose gel electrophoresis (AGE). Of the RNA samples from the twenty goats in the two groups, three were randomly chosen from each group for the construction of cDNA libraries for mRNA and sRNA sequencing. Library preparation, high-throughput RNA-seq, and data analyses were conducted as previously described [5]. High-quality clean reads were aligned with HISAT2, and gene quantification was carried out using StringTie software (v2.2.1) [13]. The Coding Potential Calculator 2 (CPC2) [14], the Coding–Non-Coding Index (CNCI) [15], and Protein Families database (PFAM) scans [16] were used to predict the coding potential of lncRNA. The intersections of transcripts that were not found to have coding potential using these three tools were selected as candidate datasets of novel lncRNA. The transcript expression levels are expressed as fragments per kilobase of transcript per million mapped read (FPKM) values. Differential expression was analyzed using DESeq2 [17], with an adjusted *p*-value of < 0.05 and |log2(fold-change)| ≥ 1 as the cut-offs for the lncRNA and mRNA. The raw sequencing dataset obtained from RNA-Seq was submitted to NCBI (PRJNA977458).

The lncRNA-target genes were identified by applying cis and trans principles to explore the effect of the prolactin levels on lncRNA. Furthermore, we performed a Kyoto Encyclopedia of Genes and Genomes (KEGG) enrichment analysis for the target genes of lncRNA using the KEGG orthology-based annotation system [18].

### 2.3. Construction of the lncRNA–miRNA–mRNA Interaction Network

We constructed an lncRNA–miRNA–mRNA interaction network where lncRNAs and protein-coding RNAs containing one or more common microRNA response elements (MREs) can competitively bind to miRNAs and regulate each other’s expression, thus allowing for an investigation of lncRNAs’ role in prolactin-regulated testicular function. Specific miRNA targets of mRNA and lncRNA were identified using the RNAhybrid (v2.0) [19] and miRanda (v3.3a) [20] software. Then, we selected the predicted target lncRNAs that had opposite expression patterns to the corresponding miRNAs as candidate target lncRNAs. Finally, the interaction network was constructed using Cytoscape_v3.9.1.

### 2.4. Isolation and Culture of Leydig Cells

The testes of cashmere goats were brought to the laboratory, and then, they were washed with PBS. Leydig cells were isolated via collagenase digestion. In brief, the testicular tissues were digested with collagenase type IV (Sigma, St. Louis, MI, USA) at 1 mg/mL and 0.25% trypsin. The digestion was terminated by adding an equal volume of DMEM/F12 medium containing 10% fetal bovine serum. The supernatant was collected via filtration through a 70 μm cell strainer. Subsequently, the filtrate was centrifuged at 1200 rpm. After that, they were washed with DMEM/F12. As previously described [21], the purity of the cells was verified using the 3β-hydroxysteroid dehydrogenase staining method, and it was found to be above 90%; thus, these cells could be used for the follow-up experiment. The Leydig cells were resuspended and seeded in cell culture plates, and they were cultured in DMEM/F12 with 10% FBS and antibiotics in a humid atmosphere at 37 °C and 5% CO_2_. The Leydig cells were passaged until they reached 85% confluence.

### 2.5. Leydig Cells Cultured with Different Prolactin Concentrations

Subsequently, we investigated the testosterone secretion levels when different prolactin concentrations were added to the cultured Leydig cells. Prolactin (ProSpec, Rehovot, Israel) was added to the culture medium to achieve concentrations of 0, 50, 100, 150, 200, and 400 ng/mL. The media and Leydig cells were collected after 24 h and 48 h of treatment, respectively.

### 2.6. Plasmid Construction and Cell Transfection

The full-length sequence of lncRWDD3 was amplified by means of PCR and then subcloned into the BamHI/EcoRI restriction sites of pCDNA3.1(+), generating pCDNA3.1(+)-lncRWDD3. An NPY1R overexpression vector (pcDNA-3.1(+)-NPY1R) was constructed by amplifying the NPY1R-coding sequence (CDS), and these were integrated into the BamHI/EcoRI sites of the pcDNA3.1(+) overexpression plasmid. The empty pcDNA3.1(+) vector was used as the control plasmid. The miR-1388-5p mimics, mimic NC, siRNA targets of the NPY1R gene (si1-NPY1R, si2-NPY1R, and si3-NPY1R), siRNA target against the lncRWDD3 gene (si1-lncRWDD3, si2-lncRWDD3, and si3-lncRWDD3), and siRNA nonspecific control (si-NC) were synthesized by Genepharma Biological. Transfection was initiated when cell confluence reached 70–80%. All of the transient transfections or co-transfections were achieved using Lipofectamine 3000 reagent (Invitrogen, Carlsbad, CA, USA). The primer sequences used are listed in Table S1.

### 2.7. Dual-Luciferase Reporter Assay

Wild-type (WT) and mutant-type (MUT) lncRWDD3 and NPY1R 3′UTR containing chi-miR-1388-5p-binding sites were designed and synthesized; they were inserted into the XhoI/NotI sites of the psiCHECK-2 luciferase reporter vector. The chi-miR-1388-5p mimic and mimic NC were purchased from Genepharma Biological (Suzhou, China). Lipofectamine 2000 was used to transfect the miR-1388-5p mimic or mimic NC in order to transfect the luciferase reporter into Leydig cells. Firefly luciferase activities were detected after 48 h of transfection with a dual-luciferase assay system.

### 2.8. Fluorescence In Situ Hybridization Assay and Immunofluorescence

Anti-digoxigenin-labeled RNAs for lncRWDD3 (5′-ACTGAGATTTAAATTTCCACTAAATAACTG-3′) and NPY1R (5′-ACACATGATGGCCACAAGCAAGTCT-3′) were bought from Anhui General Biosystems. Leydig cells were seeded and cultured on coverslips placed in 48-well plates. Pre-hybridization, hybridization, and washing were conducted. Nuclear backstaining was conducted with 4′,6-diamidino-2-phenylindole (DAPI, Beyotime, Shanghai, China). DAPI fluoresced blue, and the probe signal fluoresced red, as observed using an inverted microscope.

Immunofluorescence was conducted on Leydig cells cultured in 6-well plates. The cells were fixed in 4% formaldehyde until they grew to 70% density, and then, they were washed three times in PBS for 5 min. The cells were infiltrated using 0.5% Triton X-100 and then blocked with goat serum for 30 min. Subsequently, blocking with 3% BSA and Anti-NPY1R Rabbit pAb (1: 800, GB113733-100; Servicebio, Wuhan, China) was carried out. The cells were incubated at 4 °C overnight. Finally, they were washed 3 times and stained with DAPI.

### 2.9. Cell Counting Kit-8 (CCK-8) Assay

The Leydig cells were cultured with medium containing different concentrations of prolactin for 24 h or 48 h in 96-well plates at an initial density of 1× 10^4^ cells per well. The final concentrations of prolactin in the media were 0, 50, 100, 150, 200, and 400 ng/mL. The cell viability was measured using a CCK8 kit (Dojindo, Kumamoto, Japan), according to the operating instructions.

### 2.10. 5-Ethynyl-2′-Deoxyuridine (EdU) Assay

Leydig cells were cultured, and prolactin was added at concentrations of 0, 50, 100, 150, 200, and 400 ng/mL. After 24 h, the cells were exposed to EdU. Afterward, the cells were fixed in 4% paraformaldehyde for 30 min at room temperature, then neutralized with 2 mg/mL of glycine solution, and permeabilized by adding 0.5% Triton X-100. A solution containing 1 × EdU was added, and the cells were incubated for 30 min. Additionally, Hoechst 33342 reaction solution was added to each well, followed by incubation for 30 min. Finally, three areas were selected at random, and the number of EdU-stained cells was determined using a fluorescence microscope (DMi8, Leica, Wetzlar, Germany).

### 2.11. Hormone Analysis of Testosterone and cAMP

The testosterone and intracellular cAMP levels were analyzed using ELISA kits (Nanjing jiancheng, Nanjing, China). The sensitivity of each assay was 0.05 ng/mL and 0.1 ng/mL, respectively, and both the intra- and inter-assay variation coefficients were below 10%. Absorbance was detected at 450 nm.

### 2.12. qRT-PCR

The total RNA was isolated using TRIzol reagent following the recommendations of the supplier. The cDNA synthesis of RNA was carried out using a TransScript One-Step gDNA Removal and cDNA Synthesis SuperMix Reagent kit. qPCR was carried out with an ABI QuantStudio 7 Flex System (Vazyme, Nanjing, China). β-Actin and U6 were used as the internal reference genes for the lncRNA, mRNA, and miRNA. Gene expression levels were determined using the 2^−∆∆Ct^ method. The primers are listed in Table S2.

### 2.13. Western Blotting

The overexpression plasmid, miRNA mimics, or siRNA was transfected into the Leydig cells in six-well plates. The cells were collected and washed, and then, they were lysed in lysis buffer. The total protein concentration was quantified using a BCA assay (Biomed, Beijing, China), and the sample proteins were detached using SDS-PAGE. Immunoblotting was conducted using standard procedures [22]. The antibodies used were as follows: anti-StAR (1:1000, #AB96637; Abcam, Waltham, MA, USA); anti-3β-HSD (1:1000, #AB75710; Abcam); anti-CYP17A1 (1:1000, #AB125022; Abcam); anti-CYP11A1 (1:1000, #AB272494; Abcam); and anti-17β-HSD (1:1000, bs-6603R; BIOS, China). Goat anti-mouse IgG (H+L) and goat anti-rabbit IgG (H+L) (1:5000, Thermo Pierce, USA) were also purchased. The bands were visualized using an ECL detection kit (SuperSignal^®^ West Dura Extended Duration Substrate, Thermo Scientific, Shanghai, China) in an X-ray room. The band signals were quantified using the AlphaEaseFC software (version 4.0, Protein Simple, Santa Clara, CA, USA). Relative protein expression was calculated based on the average intensity of bands relative to that of β-actin bands.

### 2.14. Statistical Analysis

The results were analyzed using an ANOVA or Student’s *t*-test in SPSS (version 21.0; Chicago, IL, USA). Data are expressed as the mean ± SEM. Each experiment was performed in triplicate. *p* < 0.05 was considered statistically significant.

## 3. Results

### 3.1. Effects of Prolactin on lncRNA Expression Profiles and Functional Enrichment Analysis of lncRNA-Target Genes

**Figure 1:**
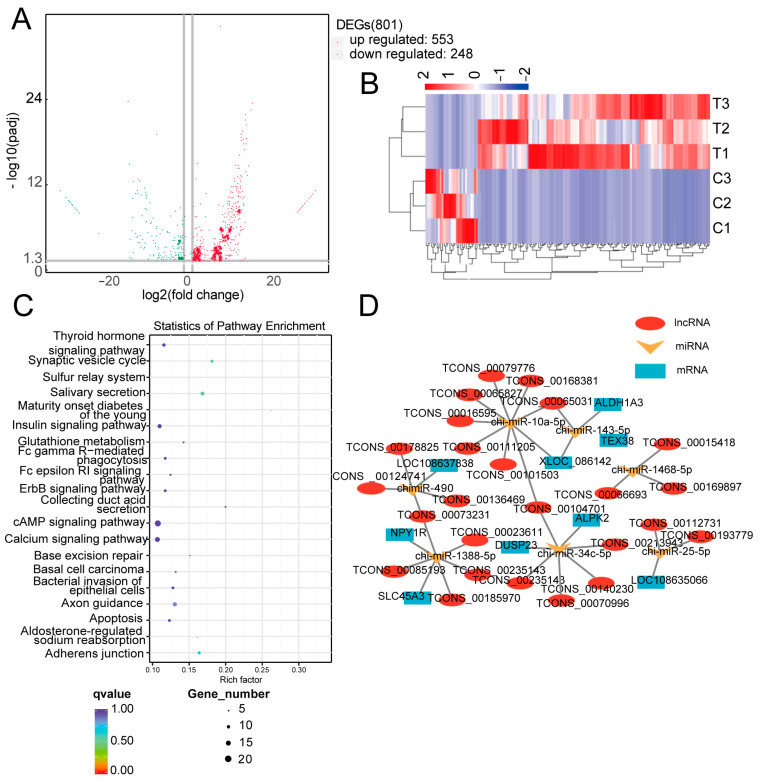
(**A**) A volcano map of DE lncRNAs. Upregulated and downregulated genes are represented as red and green dots, respectively. (**B**) Hierarchical clustering heat analysis of the differentially expressed lncRNAs. (**C**) A KEGG pathway enrichment map of the target genes of DE lncRNAs. (**D**) The lncRNA–miRNA–mRNA network.

A total of 801 (553 upregulated and 248 downregulated) differentially expressed (DE) lncRNAs were identified with an adjusted *p*-value of < 0.05 and |log2 (fold-change)| ≥ 1 (Figure 1A) in the control group vs. the prolactin inhibition group. Next, the target genes of the lncRNAs were predicted, and then, a KEGG enrichment analysis was conducted. We listed the top 20 KEGG pathways (Figure 1C), and we noticed that many of them were related to steroid hormone biosynthesis, cellular signaling, and cell proliferation/apoptosis, including the cAMP, calcium, insulin, thyroid hormone, and apoptosis signaling pathways. Details of the lncRNAs and miRNAs are presented in Tables S3 and S4.

### 3.2. LncRNA–miRNA–mRNA Interaction Network

The mRNA expression profile was presented in a previous study [5]. A total of 142 (91 upregulated and 51 downregulated) differentially expressed genes were identified in the control vs. prolactin inhibition groups (adjusted *p*-value < 0.05 and |log2(fold-change)| ≥ 1; Table S5). To further understand the biological roles of these lncRNAs, we constructed lncRNA–miRNA–mRNA interactions in accordance with ceRNA theory by combining the results of the screening for differential lncRNAs and miRNAs. In the lncRNA–miRNA–mRNA regulatory network, 9 mRNAs were found to be potentially regulated by 39 lncRNA–miRNA interaction pairs, covering 29 DE lncRNAs and 8 miRNAs. From the ceRNA network, a novel lncRNA (i.e., TCONS_00023611, named lncRWDD3 based on lncRNA cis-target genes for convenience) was predicted to bind to miR-1388-5p in order to regulate NPY1R expression and testosterone synthesis (Figure 1D). The qPCR results demonstrate that the lncRNA, miRNA, and mRNA (Table S6) expression profile was congruent with the RNA-Seq results.

### 3.3. Identification of the Testicular Leydig Cells and Fluorescence In Situ Hybridization (FISH) of lncRWDD3 and NPY1R

**Figure 2:**
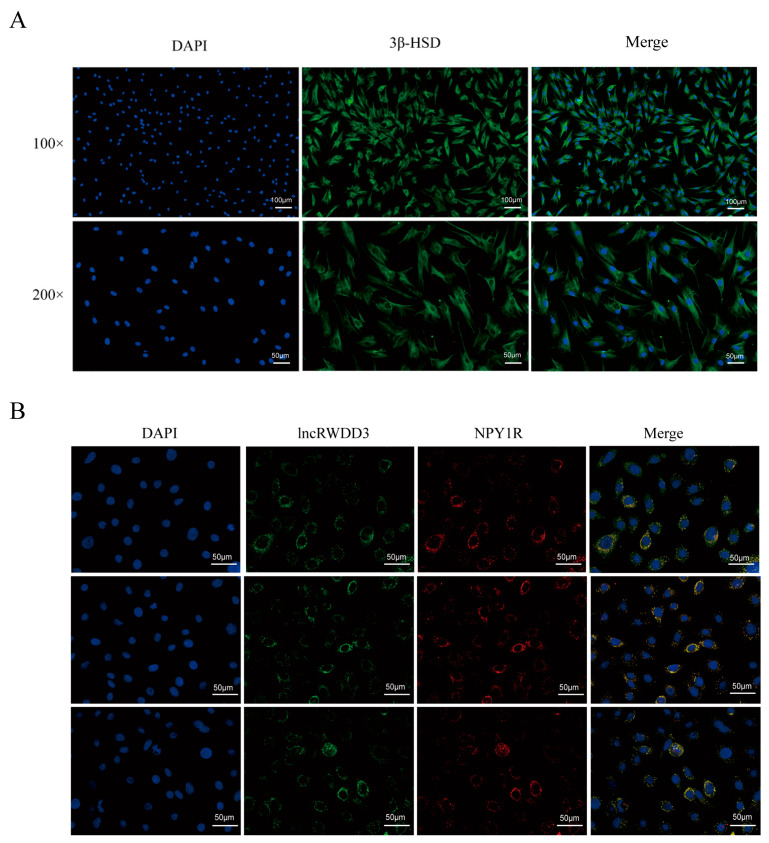
(**A**) Identification of the cultured testicular stromal cells of cashmere goats. (**B**) Expression characteristics of LncRWDD3 and NPY1R in Leydig cells. The blue fluorescence is DAPI, the green fluorescence is lncRWDD3, and the red fluorescence is NPY1R.

We isolated the goat testicular Leydig cells and characterized them by examining the immunofluorescence of 3β-HSD. The purity of the isolated primary cells reached more than 90%; thus, they could be used for experiments (Figure 2A). Subsequently, FISH revealed that lncRWDD3 and NPY1R were mostly expressed in the cytoplasm (Figure 2B).

### 3.4. The Testosterone Secretion Levels of Leydig Cells at Different Prolactin Concentrations

**Figure 3:**
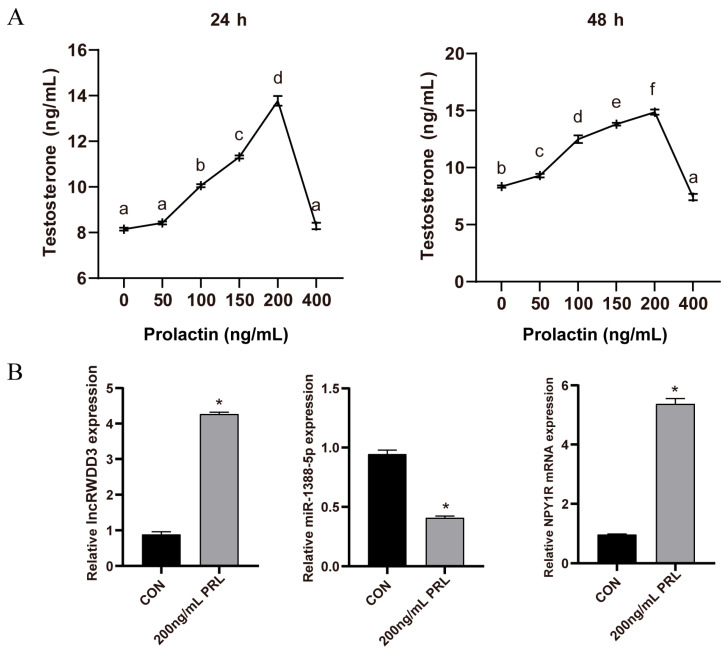
(**A**) The testosterone secretion by the Leydig cells cultured with different concentrations of prolactin. (**B**) The effect of prolactin on the expression of lncRWDD3, miR-1388-5p, and NPY1R in the Leydig cells. The results are presented as the mean ± SEM of three individual experiments. * or ^a,b,c,d,e,f^ different lowercase letters indicate significant differences (*p* < 0.05).

The testosterone secretion by Leydig cells cultured with different prolactin concentrations was examined at 24 h and 48 h (Figure 3A), and it was found that the addition of 200 ng/mL of prolactin achieved the highest levels of testosterone secretion. Then, we found that the RNA expression levels of lncRWDD3 and NPY1R were elevated. Compared to the 0 ng/mL prolactin group, miR-1388-5p was decreased in the 200 ng/mL prolactin group (Figure 3B), which is consistent with the regulatory relationships of the “ceRNA hypothesis”.

### 3.5. LncRWDD3 Promotes Testosterone Synthesis by Leydig Cells

**Figure 4:**
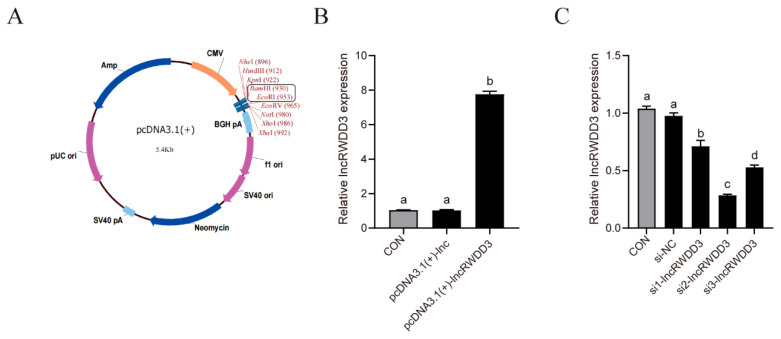
(**A**) The examination of lncRWDD3 overexpression and knockdown efficiency. (**A**) The overexpression vector of lncRWDD3. (**B**) The transfection efficiency of the overexpression vector lncRWDD3. (**C**) The transfection efficiency of lncRWDD3 knockdown. ^a,b,c,d^ Different letters indicate significant differences.

We designed overexpression vectors (pcDNA3.1(+)-lncRWDD3, Figure 4A), small interfering RNAs (si1-lncRWDD3, si2-lncRWDD3, and si3-lncRWDD3), and corresponding negative control vectors to further investigate the role of lncRWDD3. Then, we proved the effective expression using qRT-PCR. It was found to be raised after transfection with the overexpression vector pcDNA3.1(+)-lncRWDD3 (Figure 4B). Additionally, si2-lncRWDD3 was most efficiently knocked down, and it was used in the following experiments (Figure 4C).

**Figure 5:**
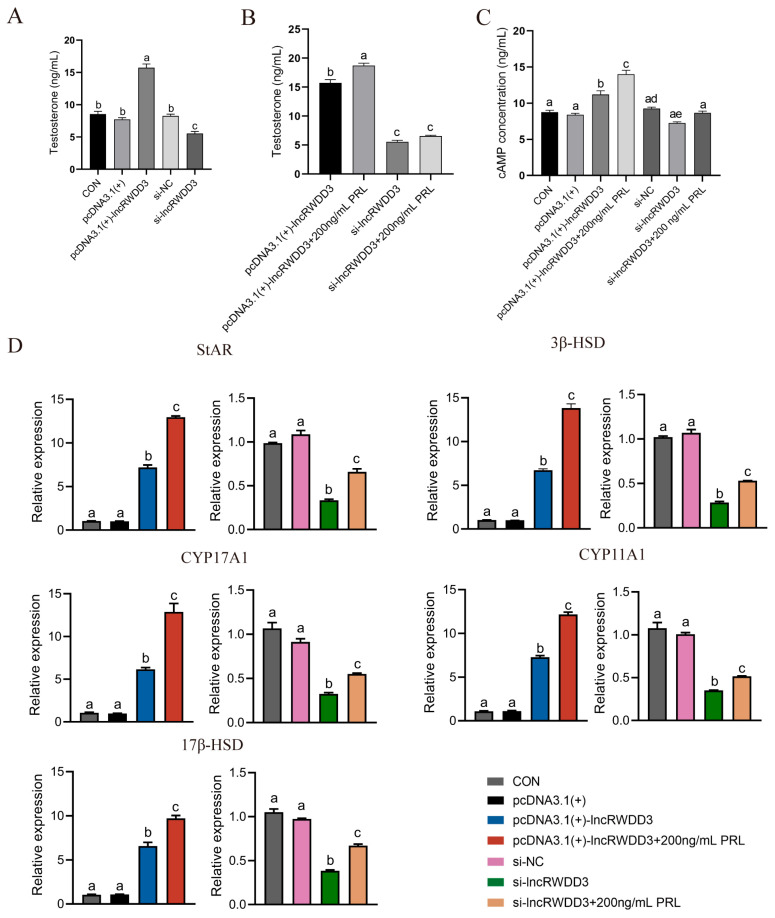
The effect of lncRWDD3 on testosterone, cAMP, and the expression of related genes in the Leydig cells. (**A**) The testosterone levels after the overexpression/knockdown of lncRWDD3. (**B**) The testosterone and (**C**) cAMP levels after the overexpression/knockdown of lncRWDD3 and the addition of PRL. (**D**) The genes’ relative expression levels of StAR, 3β-HSD, CYP17A1, CYP11A1, and 17β-HSD. ^a,b,c,d,e^ Different lowercase letters indicate significant differences (*p* < 0.05).

An ELISA indicated that the testosterone and intracellular cAMP levels of the Leydig cells were significantly enhanced by the overexpression of lncRWDD3. Contrary results were obtained for si-lncRWDD3 (Figure 5A,C). Furthermore, the testosterone and intracellular cAMP levels in the pcDNA3.1(+)-lncRWDD3 + 200 ng/mL prolactin group were significantly higher than those in the pcDNA3.1(+)-lncRWDD3 group, and the lncRWDD3 interference group showed a trend toward increased testosterone after 200 ng/mL of prolactin was added, which suggests a facilitating effect of PRL on testosterone secretion (Figure 5B). Additionally, using RT-qPCR, we discovered that, after lncRWDD3 overexpression, the expression of testosterone synthesis-related mRNAs was elevated. In contrast, lncRWDD3 interference significantly downregulated the related mRNA levels (Figure 5D). Similarly, the decrease in the testosterone-synthesis-related mRNA levels in the lncRWDD3 interference group was mollified after the addition of 200 ng/mL of prolactin (Figure 5D). In conclusion, lncRWDD3 demonstrated the biological function of promoting testosterone secretion in Leydig cells.

### 3.6. LncRWDD3 Acts as a Molecular Sponge by Binding to miR-1388-5p

**Figure 6:**
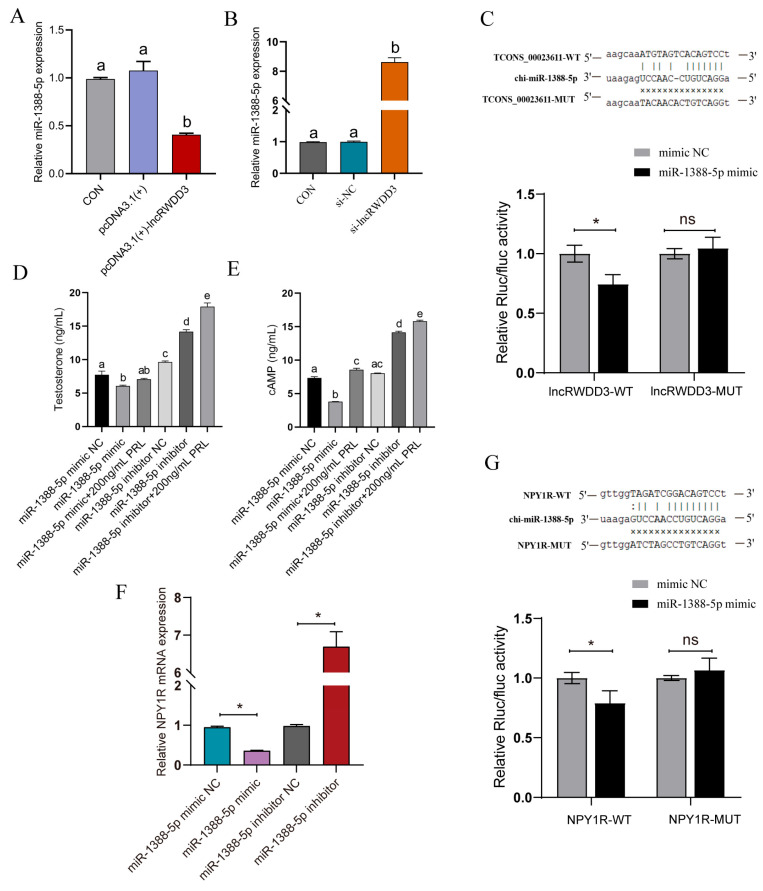
Validation of the targeting relationship between lncRWDD3 and miR-1388-5p, miR-1388-5p, and NPY1R. (**A**,**B**) The relative miR-1388-5p expression of lncRWDD3 knockdown and overexpression. (**C**) Schematic depicting the interactions of miR-1388-5p with WT lncRWDD3 and MUT lncRWDD3, and the dual-luciferase reporter gene system for verifying targeting relationships. Effects of the (**D**) overexpression and (**E**) knockdown of miR-1388-5p and the addition of PRL on the testosterone and cAMP levels in the Leydig cells. (**F**) Effect of the overexpression/knockdown of miR-1388-5p on NPY1R gene expression. (**G**) Schematic depicting the interactions of miR-1388-5p with WT NPY1R and MT NPY1R. The dual-luciferase reporter gene validation of the miR-1388-5p-targeting relationship with NPY1R. * or ^a,b,c,d,e^ different lowercase letters indicate significant differences (*p* < 0.05). ns means no significance.

LncRNAs have been shown to act as molecular sponges that regulate miRNAs, which, in turn, affects downstream target gene expression. According to the lncRNA–miRNA–mRNA interaction network analysis, miR-1388-5p is the central element and directly targets NPY1R. Therefore, we predicted that lncRWDD3 can bind to miR-1388-5p in Leydig cells, thereby acting as a molecular sponge. Firstly, we confirmed the effect of lncRWDD3 interference or overexpression on miR-1388-5p expression, the results of which revealed that lncRWDD3 overexpression downregulated miR-1388-5p expression, whereas lncRWDD3 interference had the opposite effect (Figure 6A,B). Afterward, the dual-luciferase reporter assay showed that miR-1388-5p suppressed the luciferase activities of the wild-type plasmid of lncRWDD3, while the mutant type of lncRWDD3 showed no apparent effect (Figure 6C). Together, these data suggest that lncRWDD3 may act as a molecular sponge by directly binding to miR-1388-5p.

### 3.7. MiR-1388-5p Suppresses the Steroidogenesis of Leydig Cells

We transfected the Leydig cells with the miR-1388-5p mimic, miR-1388-5p inhibitor, or related negative control (mimic NC and inhibitor NC), and we then detected the testosterone and cAMP levels using an ELISA. The results showed that the Leydig cells transfected with the miR-1388-5p mimic demonstrated inhibited cAMP and testosterone levels compared with the miR-1388-5p mimic NC-transfected Leydig cells, which were alleviated via the addition of 200 ng/mL of prolactin (Figure 6D,E). The overexpression of miR-1388-5p repressed NPY1R mRNA expression, whereas the knockdown of miR-1388-5p significantly enhanced NPY1R expression (Figure 6F). Recombinant plasmids of the wild-type and mutant NPY1R sequences were constructed using psiCHECK as a vector to determine whether there was a target-binding relationship between miR-1388-5p and NPY1R. Transfection with miR-1388-5p mimics significantly reduced the luciferase activity of the wild-type plasmids, though the mutant plasmids showed no significant influence according to the findings (Figure 6G). These findings suggest that miR-1388-5p can directly target NPY1R and negatively regulate its expression.

### 3.8. NPY1R Is Related to Testosterone Synthesis and Gene Expression

**Figure 7:**
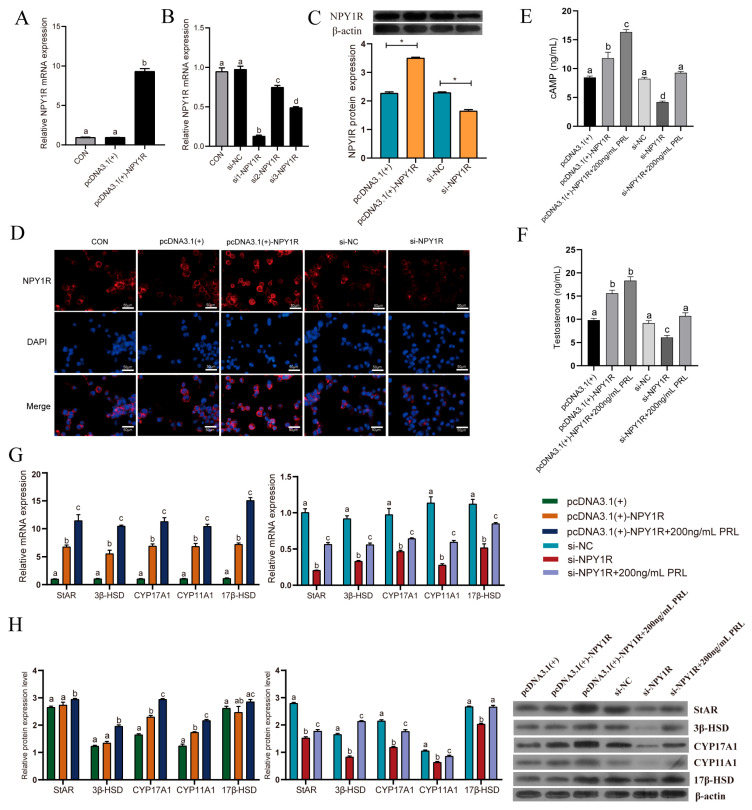
Examination of the NPY1R overexpression/knockdown efficiency and the influence of NPY1R on the expression of testosterone synthesis. Relative mRNA expression levels after the (**A**) overexpression and (**B**) knockdown of NPY1R. (**C**) The protein expression levels and (**D**) immunofluorescence staining of NPY1R. The overexpression/knockdown of NPY1R affected the related gene expression of (**E**) testosterone levels, (**F**) cAMP levels, (**G**) the mRNA, and (**H**) the protein. Scale bar = 50 µm. * or ^a,b,c,d^ different lowercase letters indicate significant differences (*p* < 0.05). The uncropped bolts are shown in Supplementary Materials.

The overexpression and interference of NPY1R were performed in vitro to investigate the potential functions of NPY1R in Leydig cells. The mRNA level was significantly raised following transfection with the pcDNA3.1(+)-NPY1R-overexpression vector (Figure 7A). We transferred three siRNAs targeting the junction site of NPY1R for transfection into the cells. Si1-NPY1R demonstrated a more potent interference effect and was chosen for follow-up experiments (Figure 7B). The NPY1R protein expression and immunofluorescence staining in the Leydig cells showed that the vector transfection was effective after the transfection of the overexpressing and interfering NPY1R vectors (Figure 7C,D).

We measured the cAMP and testosterone levels, as well as the related gene expression, to determine whether NPY1R influences the testosterone levels in Leydig cells. The results revealed that the overexpression of NPY1R increased the cAMP and testosterone levels (Figure 7E,F); meanwhile, testosterone-synthesis-related mRNA expression levels were found to be significantly upregulated (Figure 7G). A Western blot analysis revealed that NPY1R overexpression notably enhanced the protein expression of CYP17A1 and CYP11A1 (Figure 7H). On the contrary, NPY1R knockdown led to significant declines in the testosterone and cAMP levels, and it also significantly downregulated the mRNA expression levels of the testosterone-synthesis-related genes (StAR, 3β-HSD, 17β-HSD, CYP17A1, and CYP11A1). Similarly, the corresponding protein levels were significantly reduced. Furthermore, the addition of PRL had a compensatory effect on the reduced testosterone levels after the knockdown of NPY1R (Figure 7E–H).

### 3.9. LncRWDD3 Overexpression Rescues the Inhibitory Effects of miR-1388-5p on Testosterone Synthesis and NPY1R Expression in Leydig Cells

**Figure 8:**
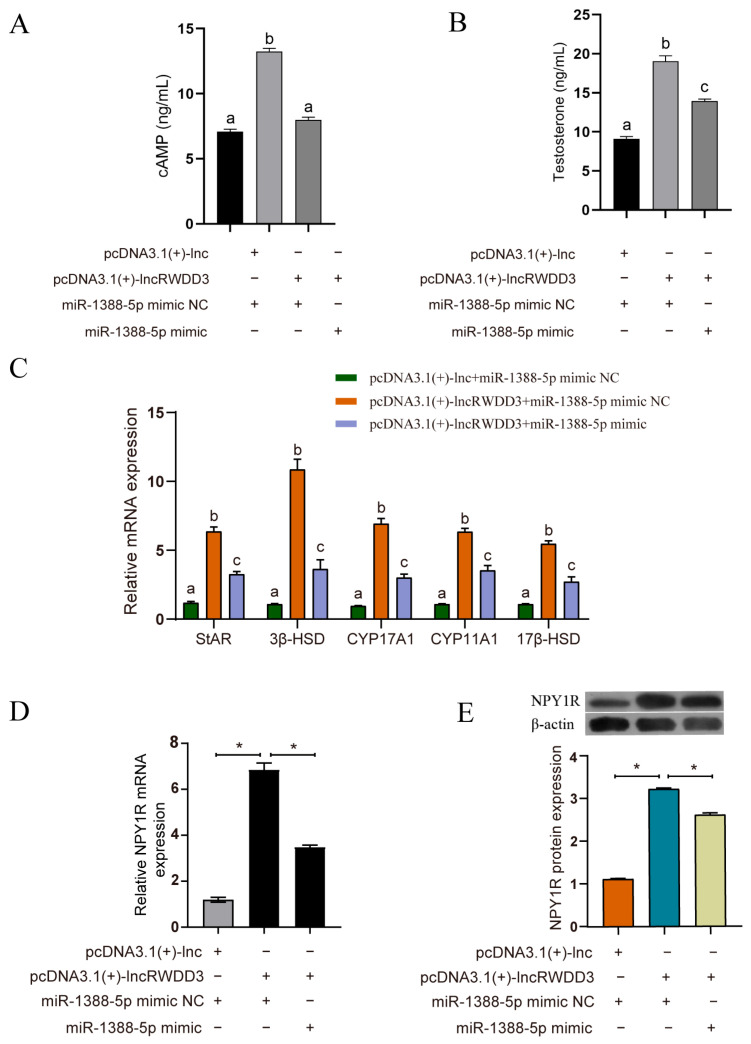
MiR-1388-5p reverses the effects of lncRWDD3 on NPY1R expression and testosterone synthesis. The (**A**) cAMP and (**B**) testosterone concentrations. (**C**) The mRNA expression levels of related genes. (**D**) The relative mRNA levels of NPY1R. (**E**) The relative protein levels of NPY1R. * or ^a,b,c^ different lowercase letters indicate a significant difference (*p* < 0.05). The uncropped bolts are shown in Supplementary Materials.

We further investigated the regulation of NPY1R expression by lncRWDD3 and the function of miR-1388-5p in the lncRWDD3/miR-1388-5p/NPY1R axis. The findings confirmed that the overexpression of lncRWDD3 significantly enhanced both testosterone and cAMP levels (Figure 8A,B). The mRNA expression levels of the steroid-hormone-synthesis-related genes StAR, 3β-HSD, 17β-HSD, CYP17A1, and CYP11A1 were also upregulated. However, the co-transfection of the overexpression of lncRWDD3 and the miR-1388-5p mimic led to a significant decrease in testosterone and cAMP levels, as well as downregulation of the expression levels of steroid-hormone-synthesis-related genes (Figure 8C). Furthermore, the overexpression of lncRWDD3 significantly promoted NPY1R gene and protein expression. The co-transfection of the overexpression of lncRWDD3 and the miR-1388-5p mimic downregulated the expression of the NPY1R mRNA and protein when compared with the overexpression of lncRWDD3 (Figure 8D,E). These results suggest that lncRWDD3 acts as a ceRNA sponge for miR-1388-5p to protect NPY1R from its attack.

## 4. Discussion

The testis plays a fundamental role in male reproduction, and proper testicular function relies on the hormones of the endocrine and paracrine pathways, whether in vivo or in vitro [23,24,25]. Our previous study demonstrated that prolactin levels have a significant effect on testicular function, including testosterone levels and testicular morphology [5]. However, the molecular mechanism by which prolactin regulates testis function remains unclear. Transcriptome technology provides valuable information on the mechanisms of the response to endocrine regulation. LncRNAs have been shown to participate in various molecular regulatory processes of mammal testicular functions [26,27]. THOR is a highly conserved testis-associated oncogenic lncRNA that can interact with insulin-like growth factor 2 mRNA-binding protein 1 (IGF2BP1), and it plays a potential role in testicular physiology processes [28]. Yang et al. identified the expression patterns of lncRNA and mRNA at the premature and mature stages of sheep testes, which indicated that PRKCD plays a critical function in spermatogenesis by interacting with the lncRNA TCONS 00863147 [29]. Furthermore, a previous study suggested a ceRNA hypothesis, where lncRNAs or circRNAs containing MREs function as molecular sponges to competitively bind to miRNAs and modulate mRNA expression [30]. We constructed a ceRNA network and analyzed the roles of lncRNAs and mRNAs in this network. Firstly, we found that the testosterone and cAMP levels in cultured Leydig cells increased with increasing prolactin concentrations up to 200 ng/mL and then decreased at higher concentrations. In addition, 200 ng/mL of prolactin was found to achieve the highest readings among all concentrations. A study in cockerels showed that, in comparison with higher levels of prolactin, Leydig cells cultured with 100 ng/mL of prolactin increased the production of testosterone, which suggests that appropriate prolactin concentrations stimulate testosterone synthesis [31,32]. Additionally, we found that the addition of 200 ng/mL of prolactin increased the expression of lncRWDD3 and NPY1R, whereas it decreased the expression of miR-1388-5p. This result indicates that prolactin has an important effect on testosterone synthesis in Leydig cells and an influence on the lncRWDD3/miR-1388-5p/NPY1R network.

NPY1R belongs to the superfamily of G-protein-coupled receptors and primarily activates or inhibits the cAMP pathway, thereby participating in testosterone synthesis [33,34,35]. In this study, we observed a candidate lncRNA (lncRWDD3) that might regulate the expression of NPY1R by targeting miR-1388-5p, wherein prolactin may regulate testicular function through lncRWDDD3/miR-1388-5p/NPY1R signaling. Leydig cells are the principal source of androgens, and they are found in the interstitium of seminiferous tubules [36]. Given the existing documentation reporting the significance of NPY1R [37,38] and the above results, we isolated the Leydig cells to further assess the moderating effect of lncRWDD3 and NPY1R. StAR regulates the rate-limiting step in steroidogenesis [39]. Studies have identified StAR as a novel target of miRNA let-7, which is modulated by lncRNA H19, and the overexpression of H19 enhances StAR expression by antagonizing let-7 [40]. Through a dual-luciferase reporter assay, our study confirmed that lncRWDD3 can adsorb miR-1388-5p to decrease its expression in Leydig cells. Meanwhile, the expression levels of genes related to testosterone synthesis, including StAR, CYP17A1, 3β-HSD, CYP11A1, and 17β-HSD, increased; consequently, the cAMP and testosterone levels increased. It has been reported that lncRNA LOC105611671 expression significantly increases during sexual maturation in Hu sheep testes and that it also regulates FGF9 expression by targeting miR-26a to facilitate testis steroidogenesis [12].

Commonly, miRNAs assemble with Argonaute (AGO) proteins and other silencing factors into miRNA-induced silencing complexes (RISCs), thereby inducing the degradation of target mRNA or inhibiting its translation [41]. Multiple studies have confirmed that miRNAs participate in steroidogenesis and spermatogenesis [42]. A study revealed that the inhibition of miR-1197-3p improves testosterone synthesis and increases PPARGC1A, StAR, CYP11A1, and 3β-HSD expression by targeting PPARGC1A, which shows that miR-1197-3p plays a crucial role in regulating testosterone secretion in the Leydig cells of goat testis by targeting PPARGC1A [43]. By generating a transgenic mouse model and populations of pre-meiotic cells and primary spermatocytes, Lukasz et al. revealed that miR-34b-5p regulates the meiosis process in germ cells by targeting Cdk6 [44]. In our study, we demonstrated that miR-1388-5p inhibits the secretion of steroid hormones in goat Leydig cells, and we also further discovered that NPY1R is a novel target of miR-1388-5p. NPY1R has been found to be expressed in the testes of humans [33], rats [38], and buffalos [37]. Comparative transcriptome analyses of immature and mature buffalo testes have shown that, during the postnatal development of male and female Leydig cells, the cAMP signaling pathway might be positively regulated to maintain testosterone function, and NPY1R has been recognized as a hub gene in the cAMP signaling pathway [37]. We found that NPY1R could regulate steroid hormone secretion in Leydig cells by improving the expression of genes relating to testosterone synthesis, which is consistent with the function of lncRWWDD3 and contrary to that of miR-1388-5p. These observations suggest that miR-1388-5p regulates the steroid hormone secretion of Leydig cells by targeting NPY1R. In addition, the overexpression of lncRWDD3 improves the synthesis of cAMP and testosterone, as well as the protein expression of NPY1R, while this effect is eliminated by miR-1388-5p mimics. The addition of prolactin improved the downregulation of testosterone synthesis caused by the knockdown of lncRWDD3 or NPY1R genes. These results reveal a novel signal transduction pathway, lncRWDD3/miR-1388-5p/NPY1R, which not only promotes the testosterone secretion of goat Leydig cells but also enriches the ceRNA network. While this study provides valuable insights, there are limitations to consider. Our findings are based on goat primary Leydig cells, and further research is needed to assess whether these results are consistent in other species. Additionally, the involvement of other regulatory pathways and interactions in testicular function needs future studies, also on implications of prolactin in reproductive health.

## 5. Conclusions

Our study identified a novel mechanism through which prolactin regulates testicular function via a ceRNA network. Specifically, we showed that lncRWDD3 acts as a molecular sponge for miR-1388-5p, thereby activating the NPY1R/cAMP pathway, which promotes steroidogenesis in Leydig cells. This finding provides new insights into how prolactin may modulate testicular function—particularly in goats—and underscores the complexity of its regulatory role. These results offer an important experimental basis for exploring the molecular pathways through which prolactin influences testicular physiology, and they provide a foundation for future research on goat reproductive health.

## Data Availability

The raw sequencing dataset obtained from the RNA-Seq was submitted to NCBI (PRJNA977458).

## Supplementary Materials

The following supporting information can be downloaded at: https://www.mdpi.com/article/10.3390/ani15131884/s1, Table S1: RNA sequence information; Table S2: List of the primers used in RT-qPCR; Table S3: lncRNA details; Table S4: miRNA details; Table S5: DE genes; Table S6: Fold difference in RNA expression between BCR and CON. The uncropped bolts are shown in Supplementary Materials.
