# Supplementary figures and images for: LncRNA RWDD3 Facilitates Leydig Cell Steroidogenesis by Regulating the miR-1388-5p/NPY1R/cAMP Pathway in Yanshan Cashmere Goats
Source: Animals (Basel). 2025 Jun 26;15(13):1884. doi: 10.3390/ani15131884 (PMC12248551; doi:10.3390/ani15131884)

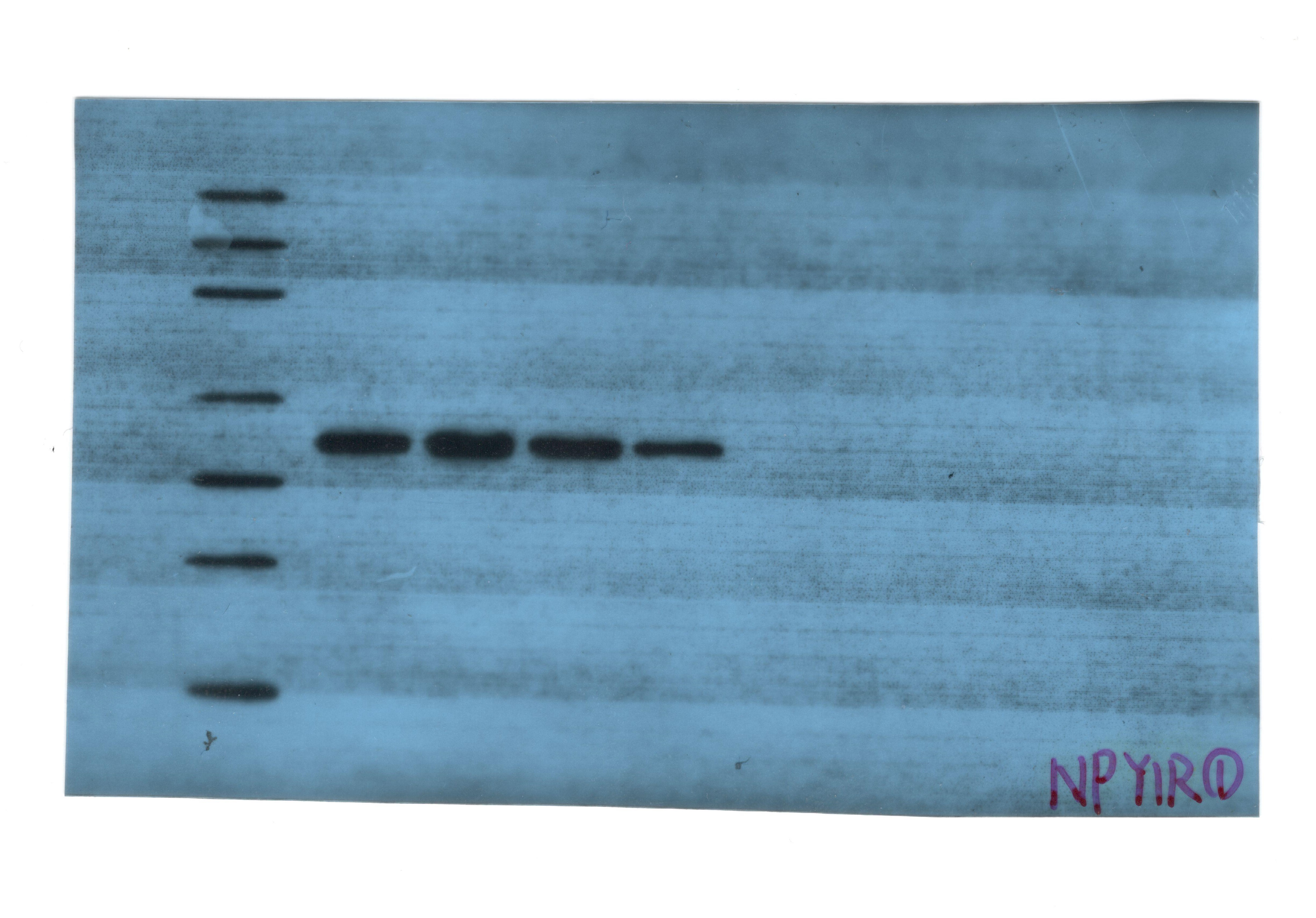

Supplement: Supplementary file 1 [file animals-15-01884-s001.zip › Western blot/Figure 7C/NPY1R.jpg]

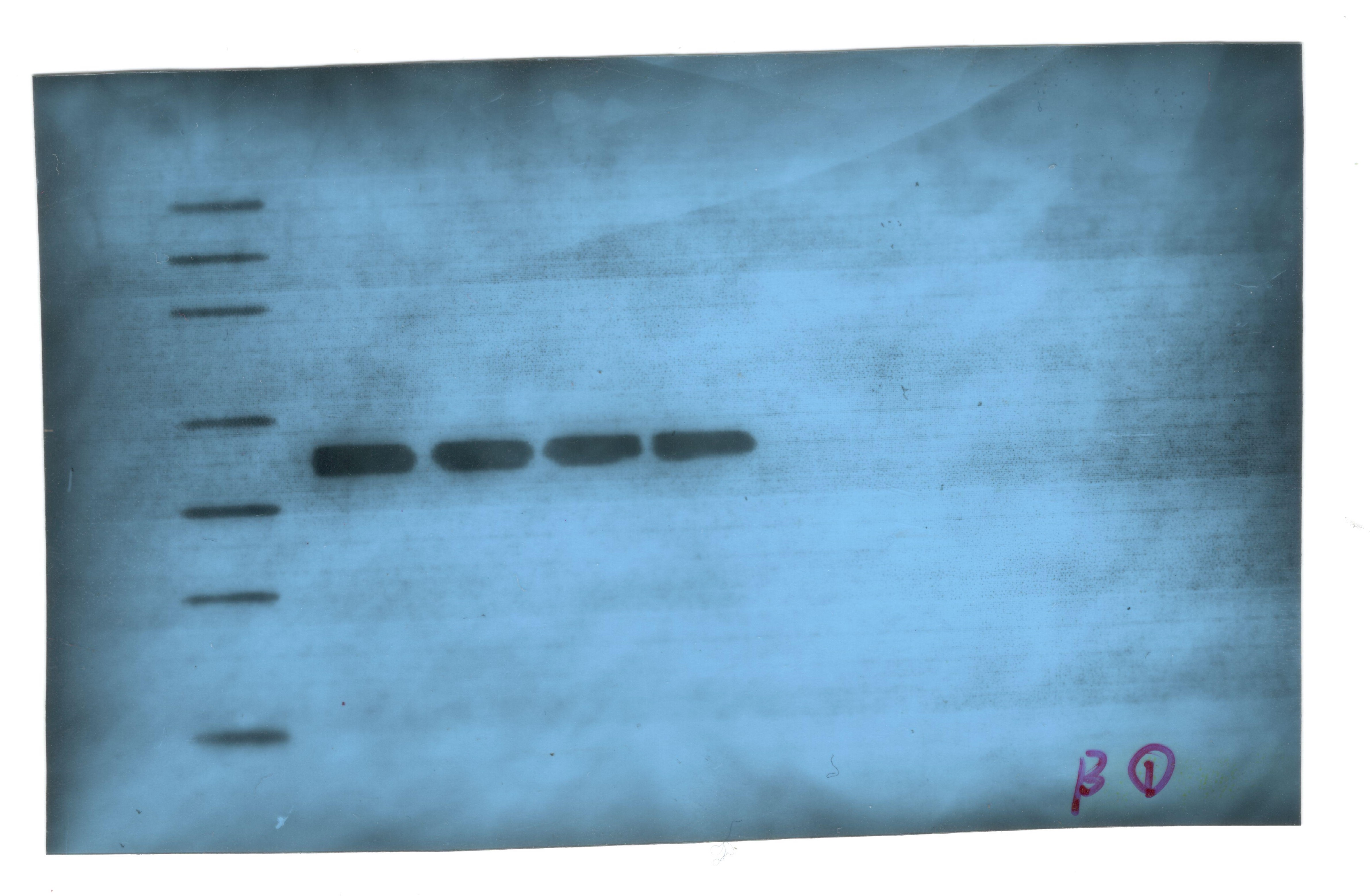

Supplement: Supplementary file 1 [file animals-15-01884-s001.zip › Western blot/Figure 7C/β-actin.jpg]

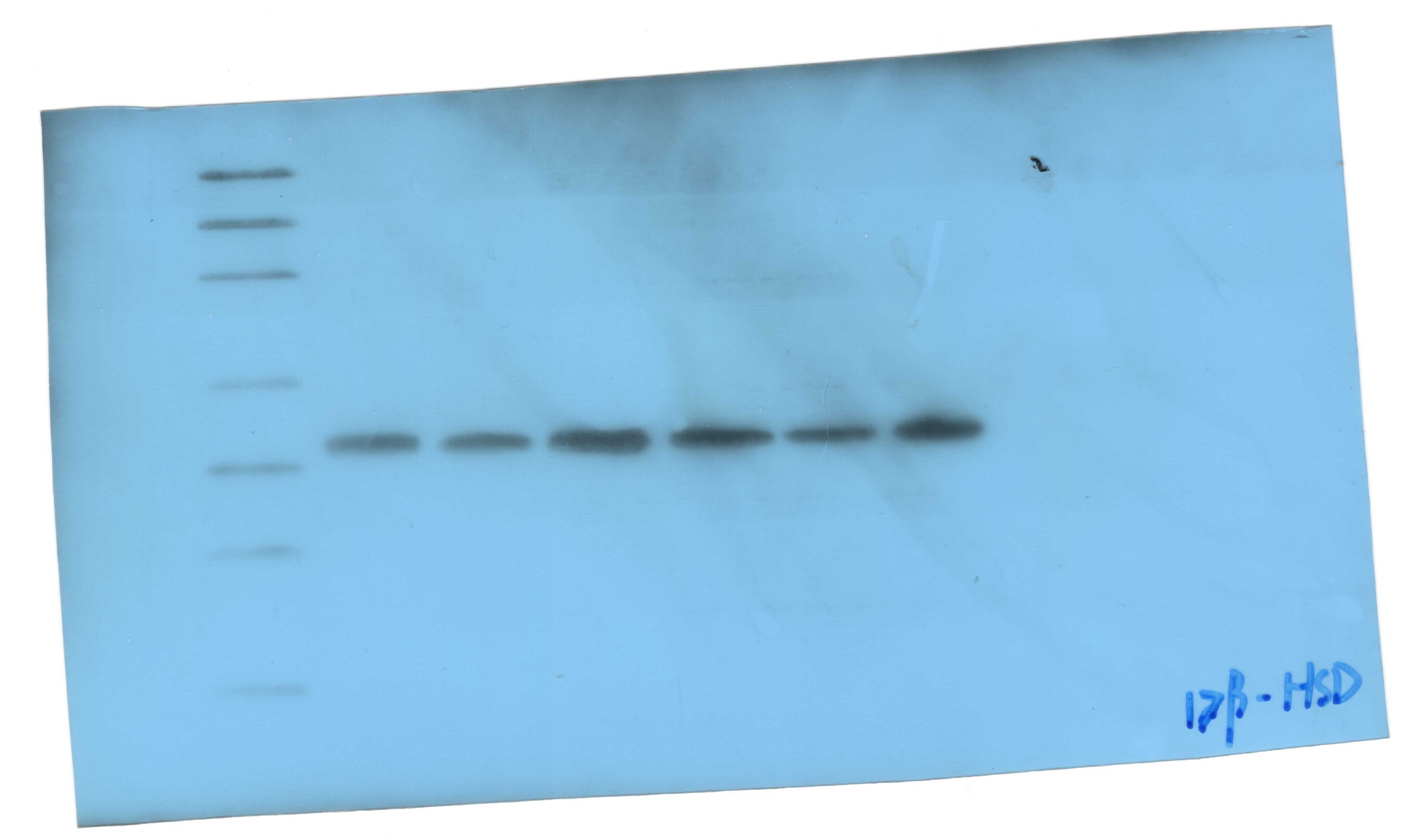

Supplement: Supplementary file 1 [file animals-15-01884-s001.zip › Western blot/Figure 7H/17β-HSD.jpg]

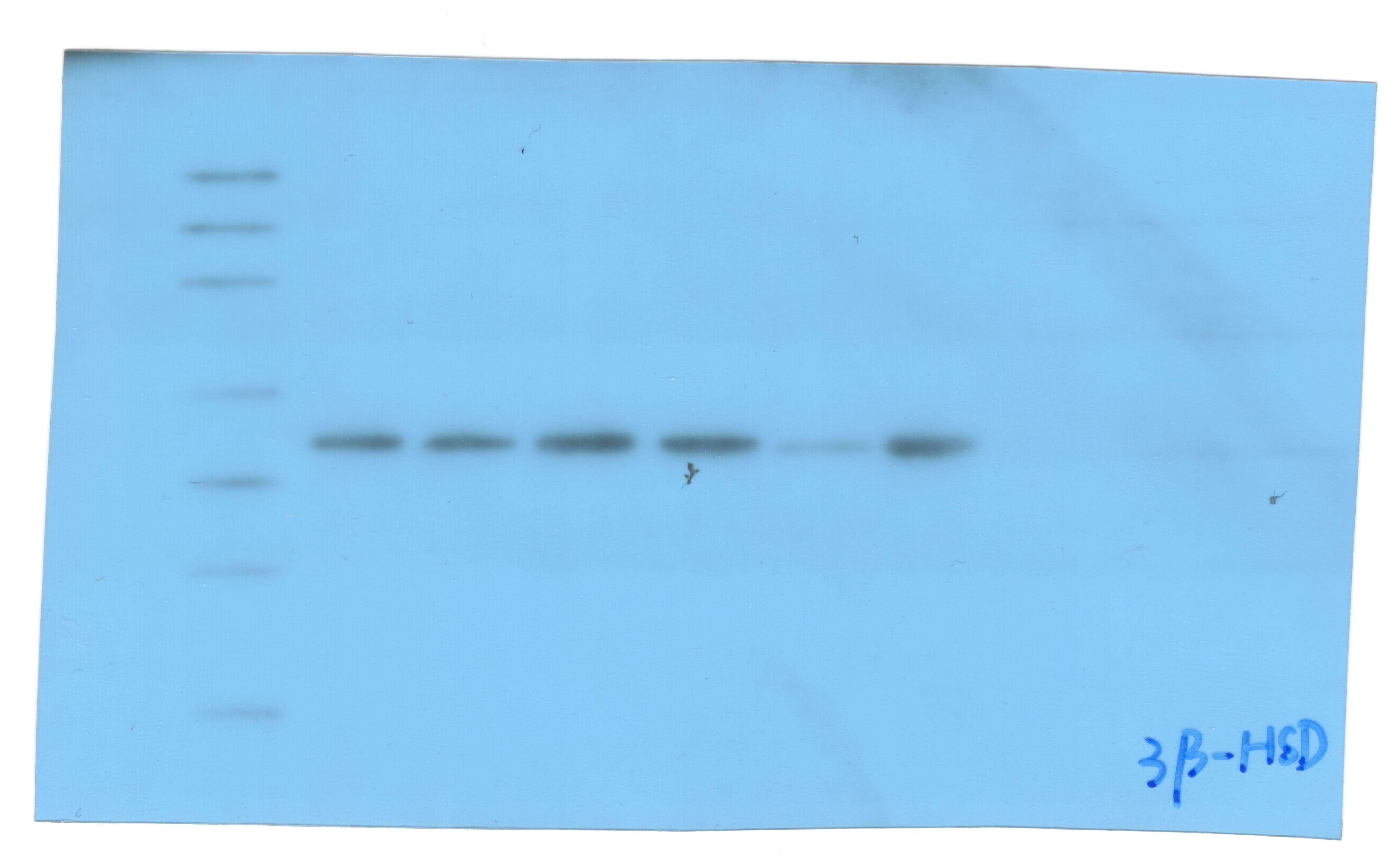

Supplement: Supplementary file 1 [file animals-15-01884-s001.zip › Western blot/Figure 7H/3β-HSD.jpg]

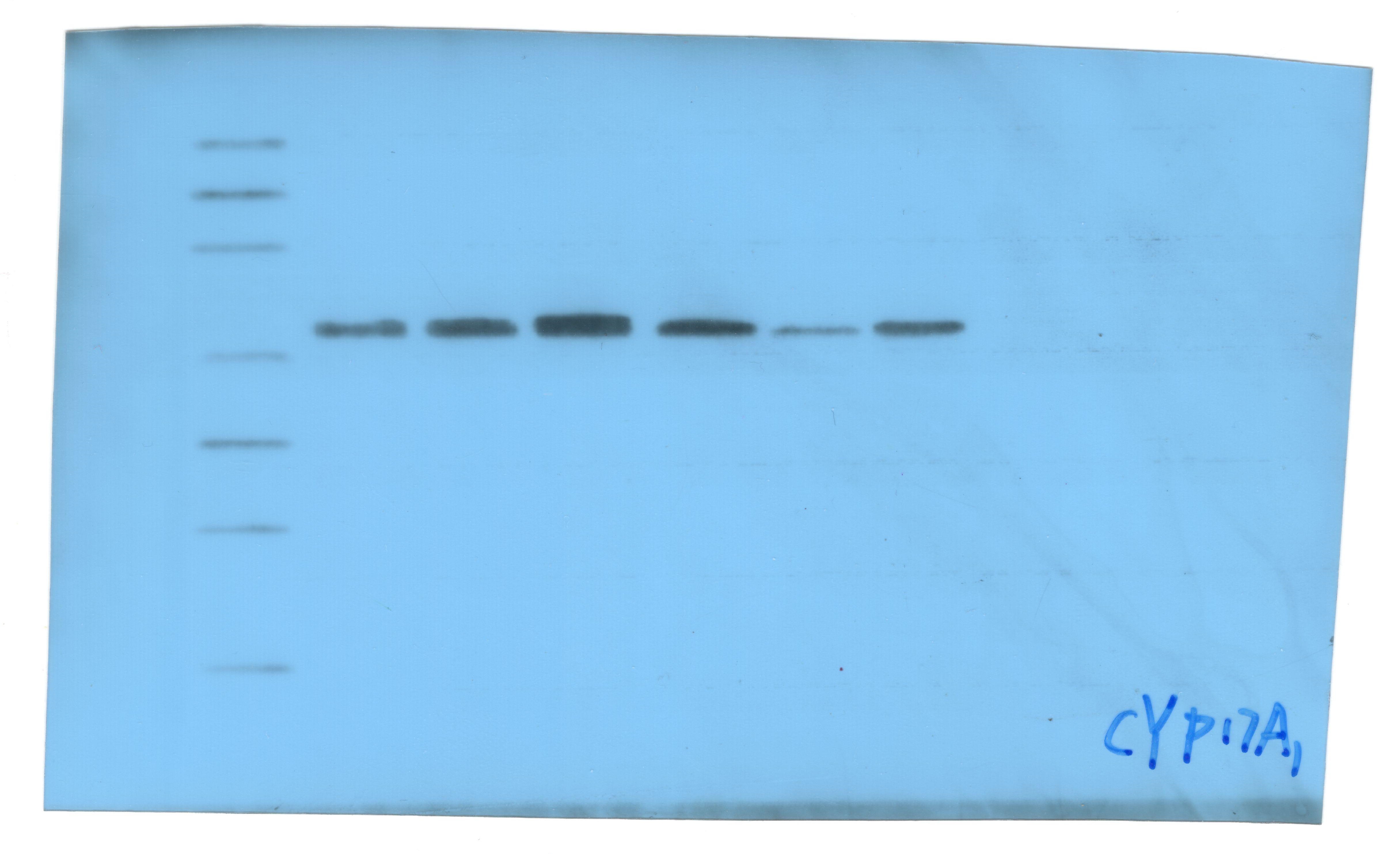

Supplement: Supplementary file 1 [file animals-15-01884-s001.zip › Western blot/Figure 7H/CYP17A1.jpg]

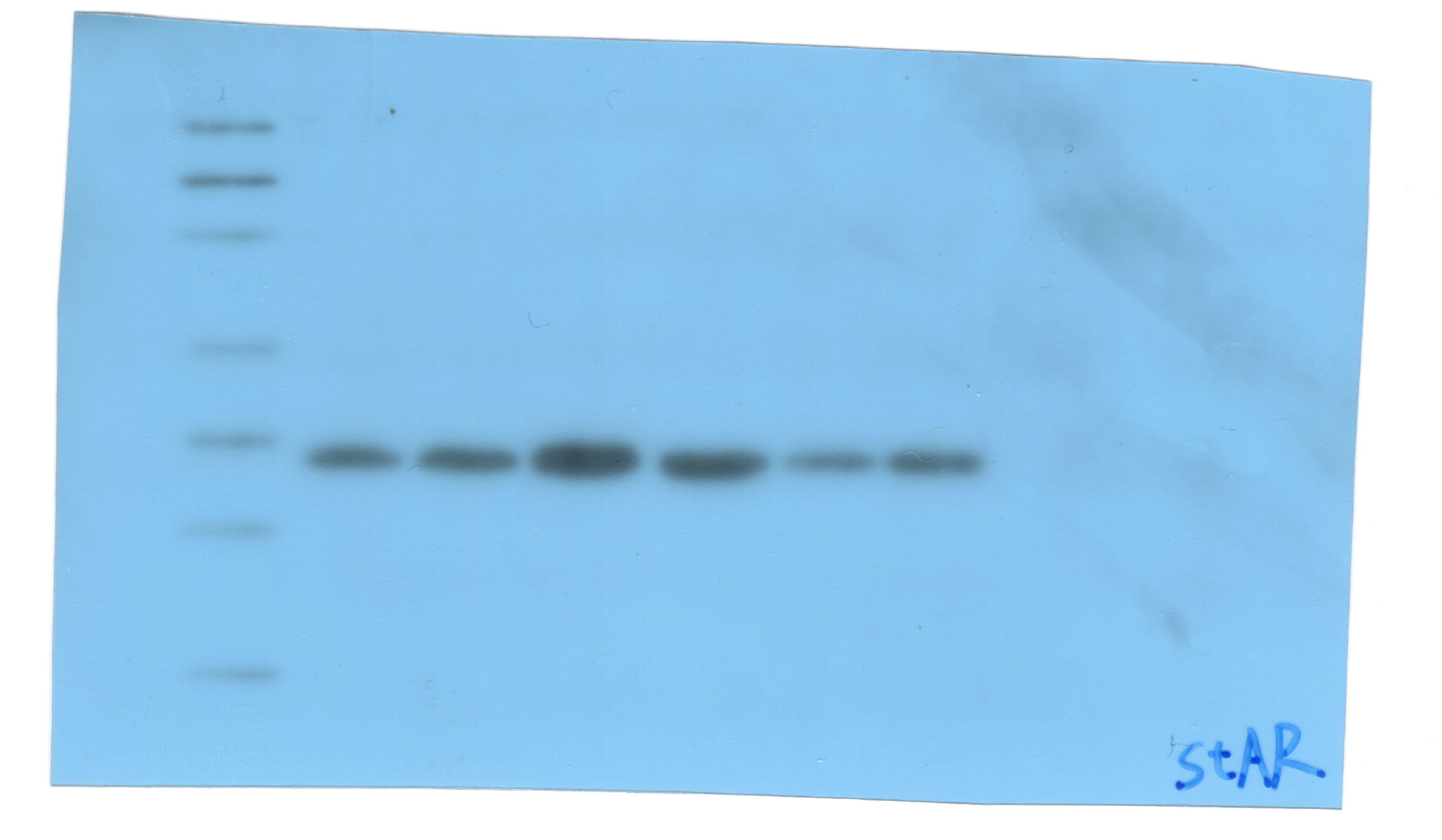

Supplement: Supplementary file 1 [file animals-15-01884-s001.zip › Western blot/Figure 7H/STAR.jpg]

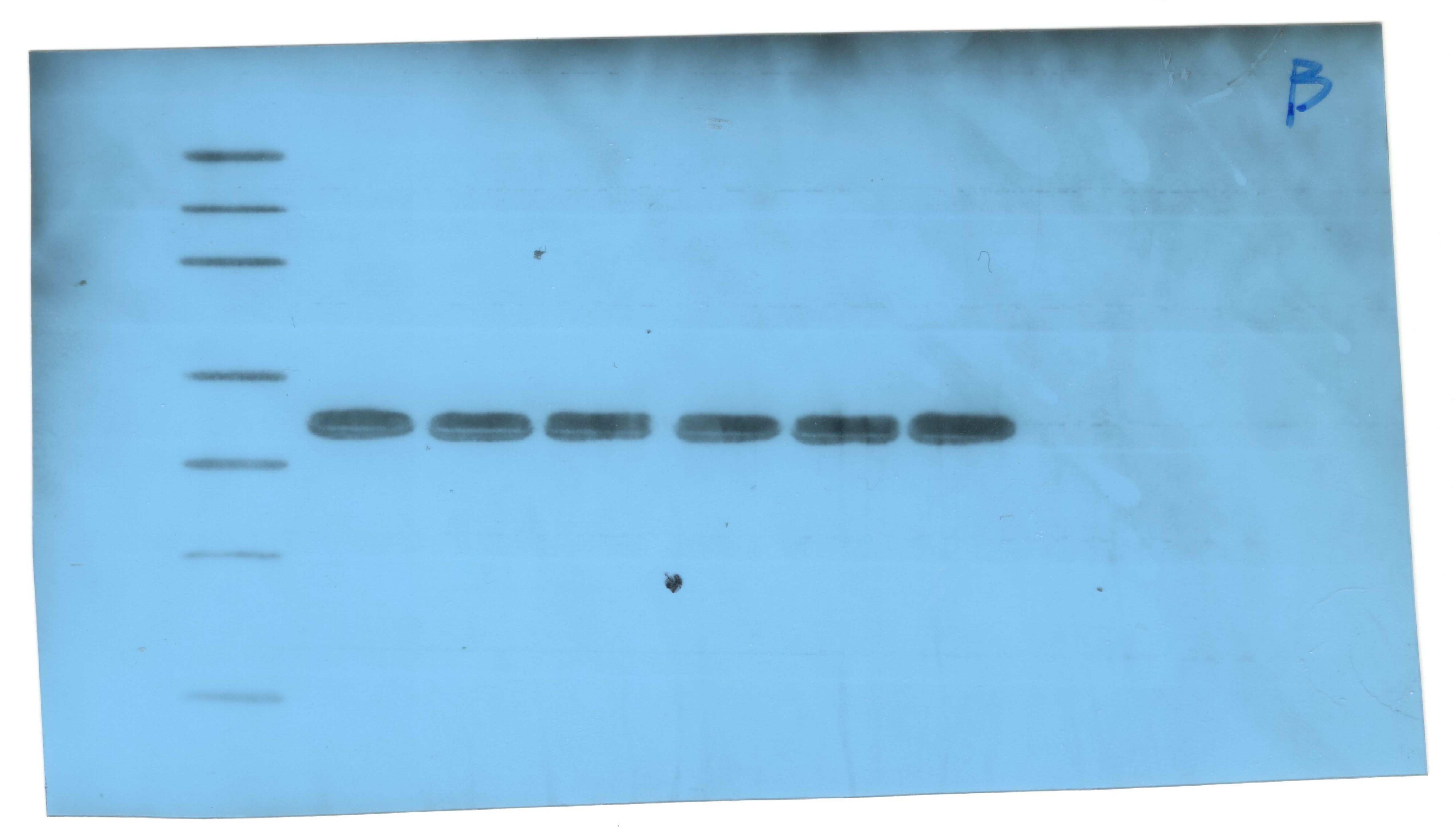

Supplement: Supplementary file 1 [file animals-15-01884-s001.zip › Western blot/Figure 7H/β-actin.jpg]

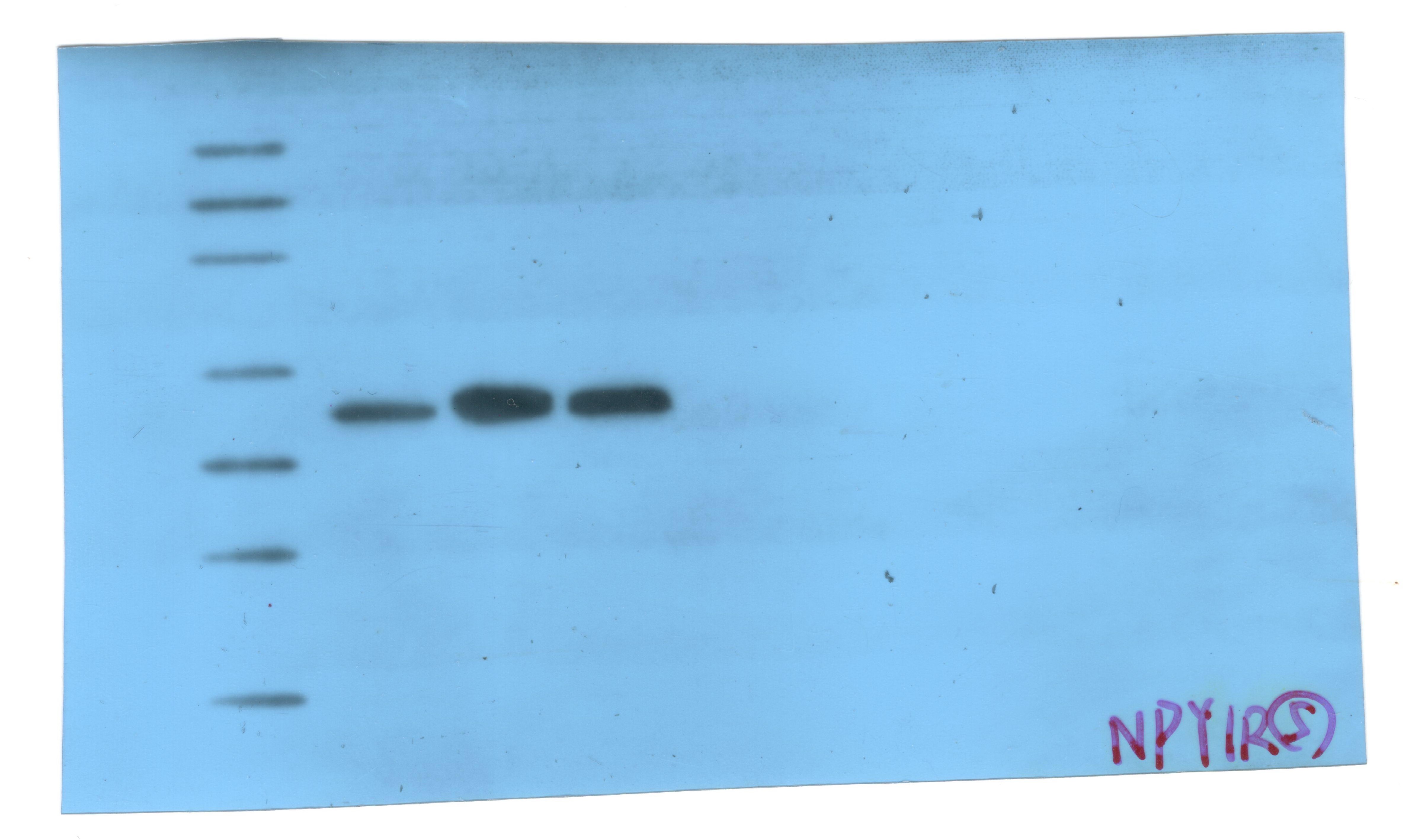

Supplement: Supplementary file 1 [file animals-15-01884-s001.zip › Western blot/Figure 8E/NPY1R.jpg]

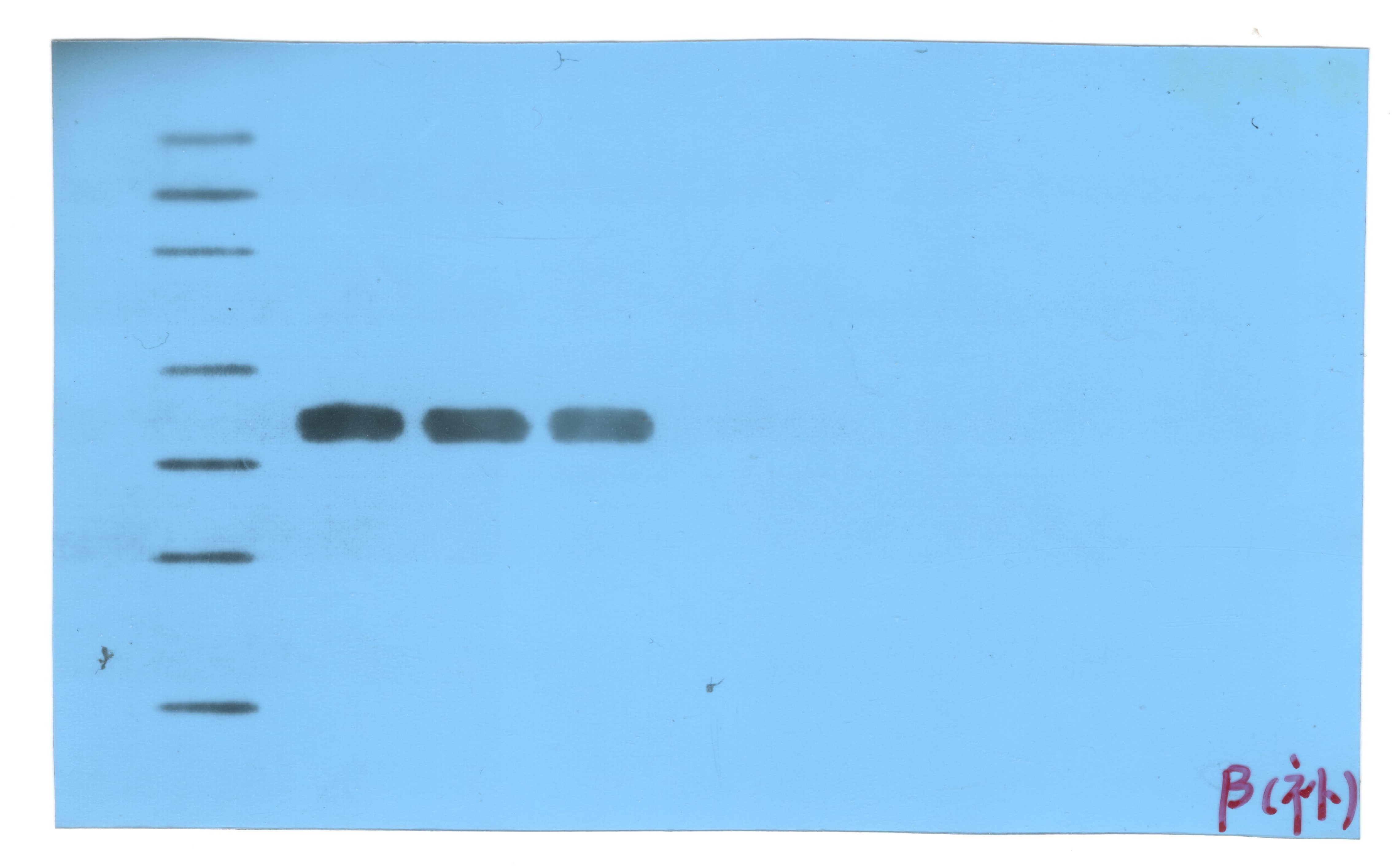

Supplement: Supplementary file 1 [file animals-15-01884-s001.zip › Western blot/Figure 8E/β-actin.jpg]
